# Evaluation of a Motion Correction Algorithm for C-Arm Computed Tomography Acquired During Transarterial Chemoembolization

**DOI:** 10.1007/s00270-020-02729-6

**Published:** 2020-12-06

**Authors:** Lena S. Becker, Marcel Gutberlet, Sabine K. Maschke, Thomas Werncke, Cornelia L. A. Dewald, Christian von Falck, Arndt Vogel, Roman Kloeckner, Bernhard C. Meyer, Frank Wacker, Jan B. Hinrichs

**Affiliations:** 1 Department of Diagnostic and Interventional Radiology, Institute for Diagnostic and Interventional Radiology, Hannover Medical School, Carl-Neuberg-Str. 1, 30625 Hannover, Germany; 2 Department of Gastroenterology and Hepatology, Hannover Medical School, Hannover, Germany; 3 Department of Diagnostic and Interventional Radiology, Johannes Gutenberg-University Medical Centre, Mainz, Germany

**Keywords:** Angiography, Transarterial chemoembolization, C-arm computed tomography, Cone beam computed tomography, Motion correction

## Abstract

**Purpose:**

The aim of this retrospective study was to evaluate the feasibility of a motion correction 3D reconstruction prototype technique for C-arm computed tomography (CACT).

**Material and Methods:**

We included 65 consecutive CACTs acquired during transarterial chemoembolization of 54 patients (47 m,7f; 67 ± 11.3 years). All original raw datasets (CACT_Org_) underwent reconstruction with and without volume punching of high-contrast objects using a 3D image reconstruction software to compensate for motion (CACT_MC_bone_;CACT_MC_no bone_). Subsequently, the effect on image quality (IQ) was evaluated using objective (image sharpness metric) and subjective criteria. Subjective criteria were defined by vessel geometry, overall IQ, delineation of tumor feeders, the presence of foreign material-induced artifacts and need for additional imaging, assessed by two independent readers on a 3-(vessel geometry and overall IQ) or 2-point scale, respectively. Friedman rank-sum test and post hoc analysis in form of pairwise Wilcoxon signed-rank test were computed and inter-observer agreement analyzed using kappa test.

**Results:**

Objective IQ as defined by an image sharpness metric, increased from 273.5 ± 28 (CACT_Org_) to 328.5 ± 55.1 (CACT_MC_bone_) and 331 ± 57.8 (CACT_MC_no bone_; all *p* < 0.0001). These results could largely be confirmed by the subjective analysis, which demonstrated predominantly good and moderate inter-observer agreement, with best agreement for CACT_MC_no bone_ in all categories (e.g., *vessel geometry*: CACT_Org_: *κ* = 0.51, CACT_MC_bone_: *κ* = 0.42, CACT_MC_no bone_: *κ* = 0.69).

**Conclusion:**

The application of a motion correction algorithm was feasible for all data sets and led to an increase in both objective and subjective IQ parameters.

**Level of Evidence:**

3

## Introduction

C-arm computed tomography (CACT) has evolved into an essential guidance tool for multiple interventions [1–10]. Various studies reported advantages of CACT over 2D digital subtraction angiography (DSA) due to improved soft-tissue resolution and the elimination of vessel superposition [1–5, 11–13]. In case of transarterial chemoembolization (TACE), CACT guidance has proven to be more robust and accurate in detecting tumor feeders than conventional DSA [14–17]. Thus, promising features of CACT are a more precise lesion detection and a more sophisticated catheter navigation for supraselective catheterizations. However, the aforementioned attributes depend on good CACT image quality. Motion artifacts such as patient movement on the table, breathing or pulsation of the heart are a potential limiting factor concerning CACT image quality, as these may lead to substantial blurring and streaking artifacts, potentially causing the loss of critical periprocedural information [1–5]. Patient movement on the angiography table or respiratory motion can be controlled by clear instructions to the patients to lay still and to perform a breath-hold for approximately 10 s (s) during the run of the C-arm. However, not all patients are able to follow these instructions and moreover, cardiac motion is inevitable. As a consequence, CACT image quality might be substantially impaired and, in some cases, repeated CACT imaging is necessary at the cost of increased radiation and contrast dose. Different attempts to optimize CACT image quality have been made [18–20]. The image reconstruction algorithm used in this study is based on the vascular reconstruction algorithm (CAVAREC, Siemens Healthcare, Forchheim, Germany), initially developed to compensate for motion artifacts in 3D cardiac imaging studies [21–23]. The aim of this retrospective single-center study was to evaluate the feasibility and the effect of a motion-compensating 3D reconstruction technique on CACT image quality, based on both objective and subjective criteria.

## Materials and Methods

### Patients

Our hospital’s Institutional Review Board approved this retrospective study. The indication for TACE was obtained by an inter-disciplinary tumor board. All TACE procedures from 01/2019 to 12/2019 (*n* = 83) were retrospectively reviewed. Of these 83 consecutive TACE procedures, 65 included a CACT (*n* = 52: 45 m, 7f; 67 ± 11.3 years). The remaining 18 were excluded due to a lack of a CACT. Patient characteristics are shown in Table 1.

**Table 1:** Patient’s demographics

Age (years)	67 ± 11.3
Gender	
Female (%)	7 (13%)
Male (%)	45 (87%)
Interventions	83
Included	65
Excluded	18
No CACT	18
Patients	52
Singular TACE	44
Multiple TACE	8
Two *x* TACE	7
Three *x* TACE	1
Hepatic artery variations	4

Patient baseline demographic and tumor characteristics

## Transcatheter Arterial Chemoembolization (TACE) Procedure

Under local anesthesia and ultrasound guidance, the right common femoral artery was assessed and a mesentericoportography was obtained. Afterward, a sequential acquisition of a hepatic angiography and a CACT was acquired to analyze tumor-feeding arteries and to plan an adequate supraselective catheter position in a breath-hold of 10 s with elevated arms, which all patients were able to perform. Contrast medium injection was performed as appropriate in accordance with our standard protocol for guide catheter (*n* = 35) or microcatheter injections (*n* = 30). Thereafter, chemoembolization with doxorubicin-loaded drug-eluting beads of 30-60 µm size (Hepa-Sphere®, Merit Medical Europe, Maastricht, the Netherlands) was performed with a microcatheter (Merit Maestro™ with Tenor™ 0.014 guidewire, Merit Medical Systems, Utah, USA) positioned in the tumor-feeding artery. Stasis in the tumor-feeding arteries delineated the endpoint of the intervention.

## Imaging and Post-processing

All procedures were performed by board-certified interventional radiologists on a monoplane, ceiling-mounted or on a monoplane, robotic-arm-mounted angiographic system (Artis Q®, ARTIS pheno®, Siemens Healthcare, Forchheim, Germany). During CACT acquisition, the X-ray source and detector, mounted on the C-arm, rotate around the patient on a circular trajectory. Image acquisition was commenced simultaneously to contrast injection. A 3D image reconstruction prototype software, developed and modified by the manufacturer (Siemens Healthcare, Forchheim, Germany), was installed on a dedicated workstation (syngo X Workplace®, Software Version VD20C, Siemens Healthcare, Forchheim, Germany) and applied to the original raw dataset. Detecting motion in all spatial directions, it was used to retrospectively modify the clinical data. By using high-density landmarks, the algorithm estimates translational, rotational and elastic deformations in the acquired images and compensates for these in the reconstruction step. Dense objects like bones, foreign material and contrast medium within the vessels can serve as landmarks, and the algorithm is tuned to optimize sparse objects like vessels. Using the same X-ray projection series as currently acquired for clinical CACT, a preliminary 3D reconstruction was generated in a first step of approximately 60 s, showing automatically segmented high-contrast objects only. In a second step of up to 90 s, manual volume punching of stationary high-contrast objects such as bones or extraneous materials was performed by a blinded radiologist, as these may interfere with the algorithm, leading to potential falsifications of motion correction in the liver. With the utilization of iterative motion estimation and compensation of a 4D deformable motion vector field, good registration of the intermediate 3D images to the 3D reference image was achieved. This 3D reference image is intermittently updated by using high contrast segmentation of the latest intermediate 3D image. The method does not require any periodicity in the motion, and all projection images are used. A preliminary version of the proposed technique was first used in cardiac imaging [21–23].

Manual performance of volume punching for bone removal enabled quantitative and qualitative comparisons between a total of three data sets: the original CACT (CACT_Org_), CACT after motion correction but without further post-processing (CACT_MC_bone_), and after motion correction in combination with prior manual bone removal (CACT_MC_no bone_). Objective quality assessment was performed using the mean image gradient intensity of prominent edges as the sharpness/blurriness metric with low values indicating blurred edges and high values indicating sharp edges to compare CACT_Org_, CACT_MC_bone_ and CACT_MC_no bon_ [24]. Image quality on the basis of subjective criteria was independently assessed by two radiologists with 8 and 1 year(s) of experience, who were blinded to the nature of the data set (CACT_Org_, CACT_MC_bone_ and CACT_MC_no bone_) and reviewed them in a random order and with a time interval of three weeks, to prevent recognition. Subjective criteria included side-by-side grading of the datasets as good (1), intermediate(2), and poor(3) for the following categories: vessel geometry, i.e., sharpness of vessel margins, overall image quality, i.e., clear visibility of vessel origins and branching, intrahepatic vessel demarcation from surrounding liver tissue. Furthermore, we performed binary grading (yes:1, no:2) of the categories: clear tumor feeder delineation, the presence of foreign material-induced artifacts (e.g., surgical clips or catheters) and need for additional imaging (e.g., repeat CACT).

For image assessment, the readers were able to use thin-sliced multiplanar reformats (MPR; slice thickness ≤ 0.49 mm) in axial, coronal, sagittal or oblique orientation and maximum intensity projections (MIP) on a 3D PACS workstation (Visage 7.1, Visage Imaging, Berlin, Germany).

## Statistical Analysis

Descriptive statistical analyses of the patient’s demographic and angiographic data are presented as mean values ± standard deviation (sd). Objective IQ was assessed by computing a no-reference image sharpness metric for each image layer of the three CACT datasets with higher values accounting for sharper, less blurred images. Thus, sharpness of 25,740 images (65 CACT with 396 images each) was analyzed and compared. Significance of differences was demonstrated by Friedman rank sum test (*p* < 0.05) and post hoc, Bonferroni-corrected Wilcoxon signed-rank test (level of significance: *p* < 0.017). Concerning subjective criteria of IQ, inter-observer agreement was calculated between CACT_Org_, CACT_MC_bone_ and CACT_MC_no bone_ by using weighted kappa test (*k*; Table 2). Kappa values ranged between poor (< 0.21) and excellent agreement (> 0.81) [25]. For each reader, differences between the subjective image quality metrics were tested via Friedman test and considered significant for *p* < 0.05. Subsequent post hoc analysis included Bonferroni-corrected Wilcoxon signed-rank test (geometry, overall IQ) and McNemar’s test for binary coded reader responses (need for further imaging, the presence of artifacts, tumor feeder demarcation; level of significance: *p* < 0.017). Statistical analysis was conducted with *R* (version 3.6.1, http://www.r-project.org with package “IRR” version 0.84.1).

**Table 2:** Inter-observer agreement for subjective criteria (kappa test)

	CACT_Org_	CACT_MC_bone_	CACT_MC_no bone_
Vessel geometry	0.62	0.54	0.69
Overall IQ	0.61	0.48	0.76
Tumor feeder visibility	0.34	0.44	0.63
Foreign material-induced artifacts	0.47	0.3	0.51

Overview of inter-observer agreement for tested subjective criteria. CACT = C-arm computed tomography; IQ = image quality; Org = original; MC = motion correction

## Results

**Fig. 1:**
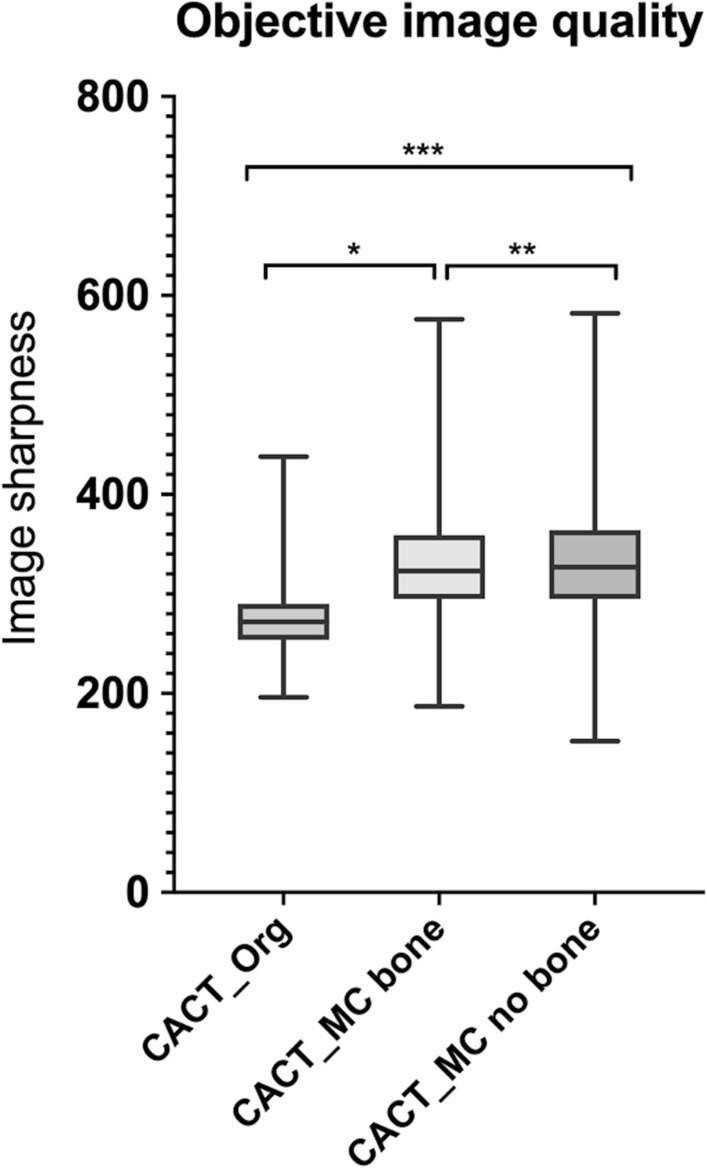
Objective image quality for the three types of reconstruction. The mean values for image sharpness increased when using the novel reconstruction algorithm. In combination with bone removal (CACT_MC_no bone_), the novel algorithm showed the highest mean value for image sharpness. Wilcoxon signed-rank test indicated significant differences between all three reconstructed CACTs (*,** and *** *p* < 0.0001)

**Fig. 2:**
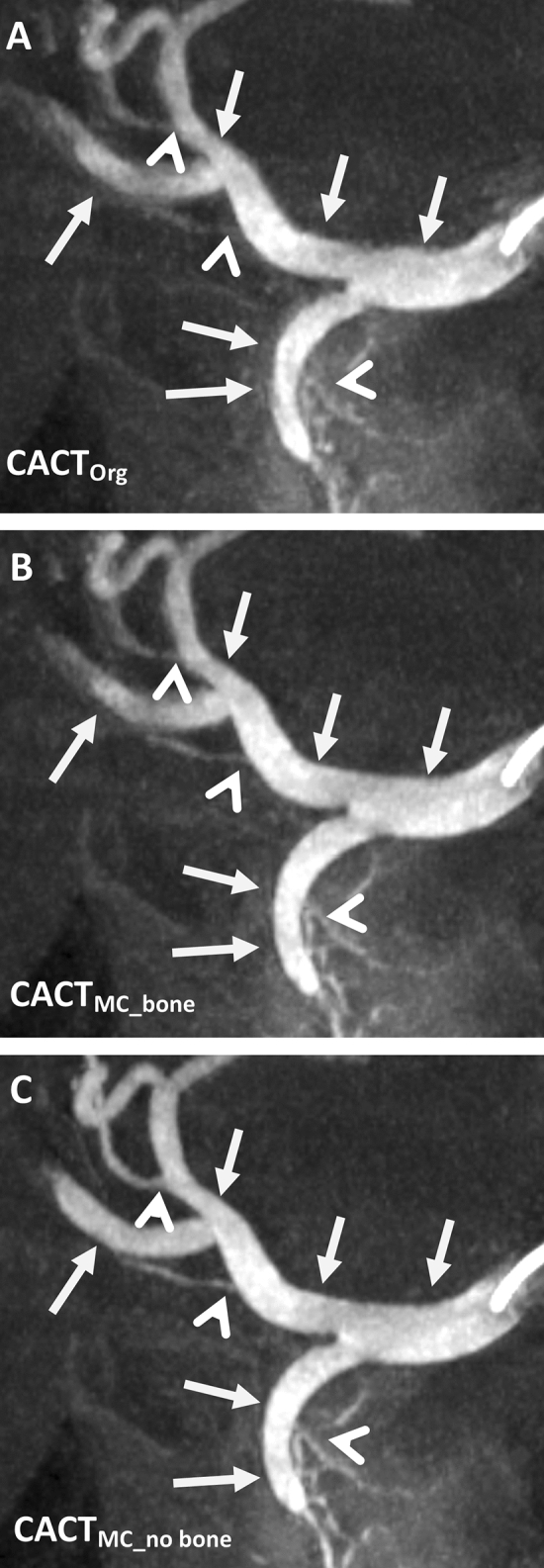
Differences in vessel geometry and overall image quality of the reconstructed CACTs of an 82-year-old male patient (coronal 10 mm maximum intensity projections). Note the initially blurry vessel borders (**A**; white arrows) becoming increasingly sharper after motion correction (**B**) and especially after motion correction application in combination with bone segmentation (**C**). Also, note the improved discernability of vessel origins from the gastroduodenal and hepatic arteries (white arrowheads)

**Fig. 3:**
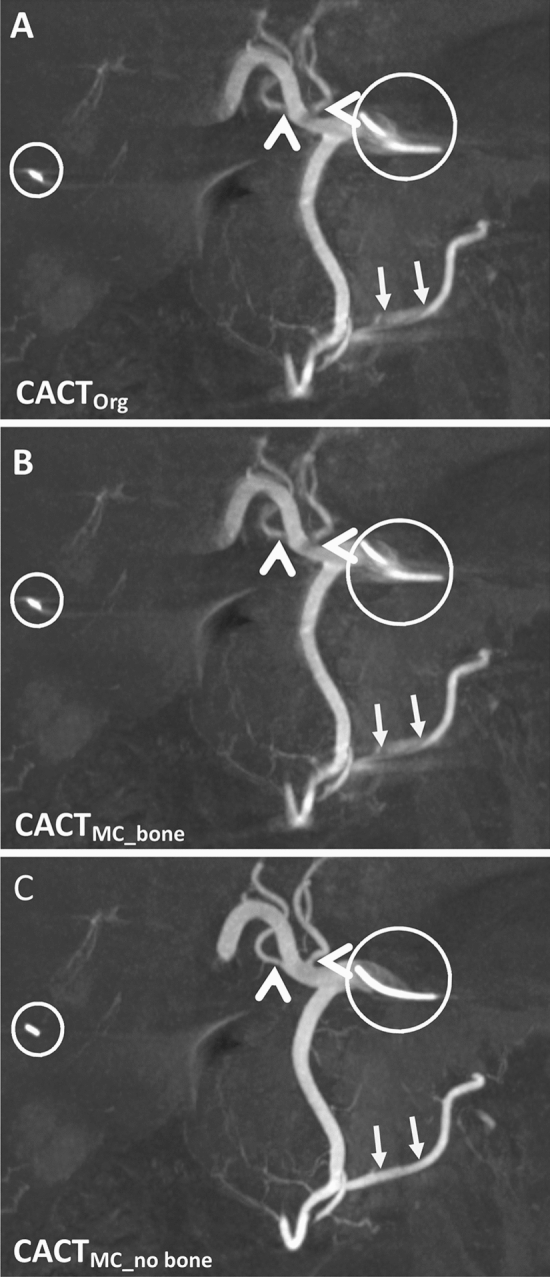
Example of evolving image quality and the more precise delineation of foreign materials in this 79-year-old male patient (coronal 10 mm maximum intensity projections). Note the discernibility of the vessels originating from the central hepatic artery, which are insufficiently depicted in CACTOrg (**A**, white arrowhead) as well as the blurry extrahepatic arteries originating from the gastroduodenal artery (**A**, white arrows). These are markedly better delineated after application of the motion correction algorithm (**B**) and especially after additional bone segmentation (**C**). Foreign material such as surgical clips or the catheter (white circle) is also noticeably sharper demarcated

**Fig. 4:**
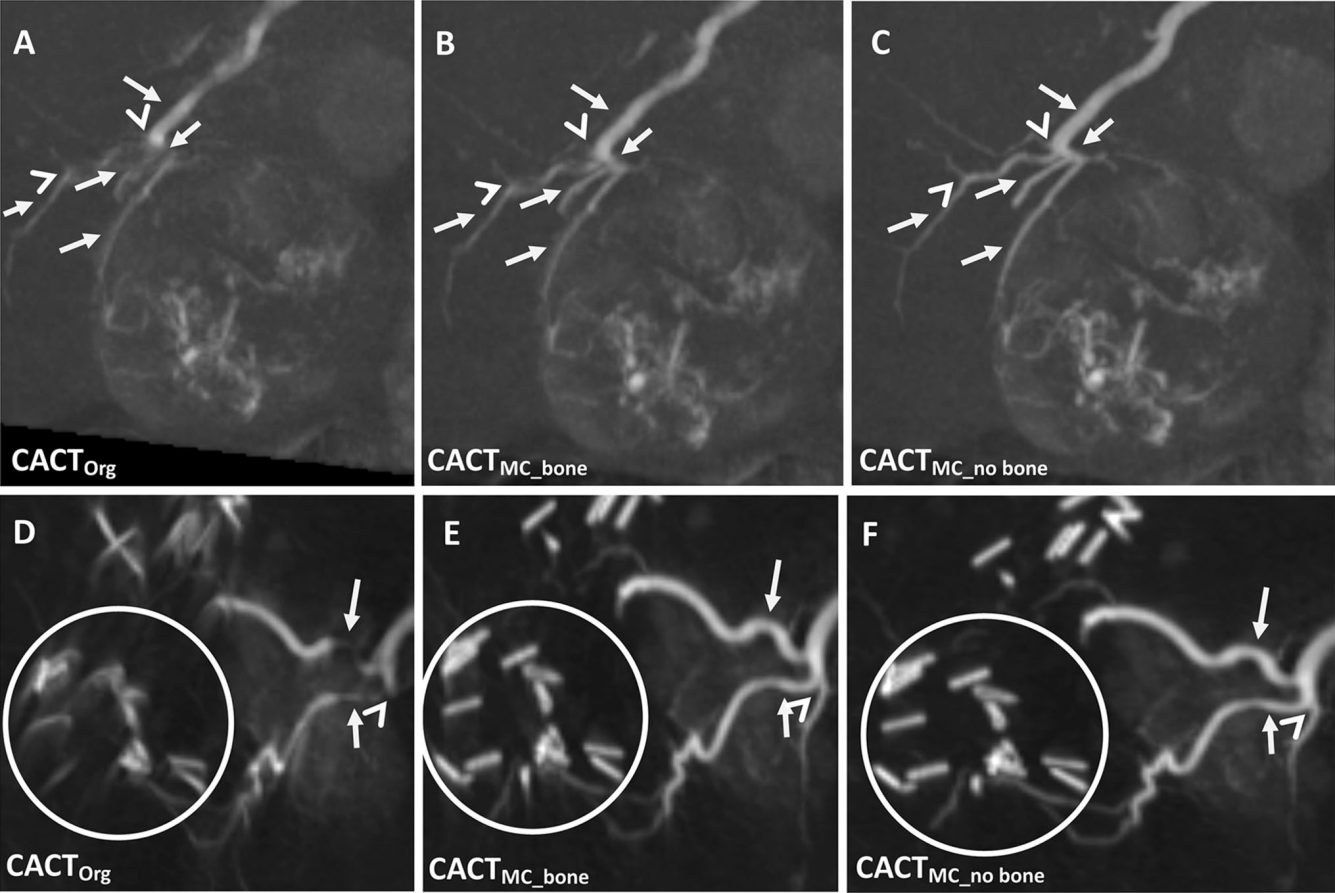
Detailed examples of CACTOrg and CACTMC_bone, CACTMC_no bone illustrating improvement of tumor feeder demarcation in a 68-year-old male (**A**–**C**) and reduction of foreign material-induced artifacts in a 47-year-old male (**D**–**F**). Vessel origins (white arrowheads), vessel course and margins are better delineated (**A**–**C**; white arrows). Surgical clip material is sharper defined (**D**–**F**; white circle). Images are shown as 10 mm coronal maximum intensity projections

Application of the motion correction algorithm proved feasible in all data sets. We analyzed 65 TACE interventions by applying objective and subjective criteria. Concerning the objective evaluation, Friedman rank-sum test (*p* < 0.0001) and consecutive Wilcoxon rank-sum test with Bonferroni-corrected alpha demonstrated significant differences between the CACT data sets and an increase in IQ after motion correction application, especially after additional bone segmentation. The sharpness metric increased from 273.5 ± 28 (CACT_Org_) to 328.5 ± 55.1 (CACT_MC_bone_) and 331 ± 57.8 (CACT_MC_ no bone_) (all *p* < 0.0001; Fig. 1). Inter-observer agreement between the two readers proved highest for CACT_MC_no bone_ (Table 2). The readers agreed that no imaging needed to be repeated. The Wilcoxon signed- rank test and McNemar’s test showed that both readers considered all post-processed CACT images, with and without bone segmentation, as significantly different from the original CACT (*p* < 0.0001), represented by a shift of the median of all subjective parameters from 2 for CACT_Org_ to 1 for CACT_MC_no bone_ (*p* < 0.0001). None of the subjective image quality parameters decreased following the application of the motion correction prototype algorithm and no undesired effects were created by the algorithm itself. Imaging examples demonstrating subjective criteria are shown in Figs. 2, 3 and 4.

## Discussion

This retrospective study of patients referred for transarterial chemoembolization demonstrates the feasibility of the application of a dedicated motion correction software on selective CACTs. The analyzed motion correction prototype software significantly improved subjective and objective IQ in all cases. The reconstruction of the dataset by the motion correction prototype algorithm used in our study took approximately 4–5 min.

In medical imaging, the purpose of IQ comprises mainly of presenting anatomical and physiological information, in order to make the best possible treatment decision for the patient. Over the last decades, there have been many attempts to develop perceptual quality metrics that are modeled after known characteristics of the human visual system [26–31]. One of the topics discussed in the imaging quality literature is the application of motion correction algorithms to address the vulnerability of CACT for motion, which included reconstruction of 4D datasets and laborious matching of 3D segmented vessels from defective CACTs to each image of the original acquisition [18–20]. In contrast to the aforementioned studies, we chose to expand the field of subjective image quality assessment by adding an image sharpness metric for analysis of objective criteria in CACTs. In order to ensure appropriate supraselective catheter positions and to avoid under- as well as overtreatment that might cause a deterioration of liver function and prevent further treatment with local and systemic therapies, increased precision and selectivity of TACE are of high interest. The observed increase in vessel origin visibility, improved demarcation of intrahepatic and tumor feeding vessels from surrounding structures, and the reduction of artifacts by foreign material might have a substantial impact on imaging guidance during intrahepatic interventions.

The benefits of CACT concerning peri-interventional guidance and diagnostic accuracy have been reported by several groups for a variety of purposes [1–5, 13, 17]. Despite these reported advantages, acquisition of CACT is associated with additional exposure to radiation and contrast media [2, 32], without the guarantee of high image quality, as this can be limited by the vulnerability of CACT to motion, potentially compromising diagnostic accuracy [6, 20, 33]. Attempts to control breathing by intraprocedural coaching of the patient before image acquisition are helpful, but often unable to completely prevent diaphragmatic motion, which has been reported in up to 50% of abdominal CACTs during liver TACE interventions and may greatly affect the liver and the depiction of liver arteries due to its anatomical proximity [19, 34]. The utilization of a motion correction algorithm showed significant improvements in the abovementioned objective and subjective parameters evaluated by us. The objective sharpness metric used in our study demonstrated a significant increase in image sharpness, growing by over 20% from the raw dataset (CACT_Org_) after the application of the motion correction algorithm (CACT_MC_bone_). Subsequent bone segmentation caused a second significant benefit of objective IQ between CACT_MC_bone_ and CACT_MC_no bone_, though less pronounced. Of note, the median grade of the subjective category “overall image quality” changed from 2 for CACT_Org_ to 1 for CACT_MC_no bone_, whereas CACT_MC_bone_ remained at a median grade of 2. This discrepancy between the increase of the objective sharpness metric and the subjective parameters might best be explained by the reader’s subjective focus on image improvements important for the interventions, which mainly consist of vessel and tumor depiction. In contrast, the objective sharpness metric analyzes the whole dataset, also addressing very peripheral structures irrelevant for the intervention, e.g., partially depicted lung parenchyma. The subjective evaluation confirmed the potential clinical impact of the objective evaluation by showing good inter-observer agreement with highest kappa values of the two readers with varying clinical experience, especially concerning overall IQ and vessel geometry for the combination of motion correction and bone segmentation. Conversely, inter-observer agreement for these categories was only moderate to good for CACT_Org_ and CACT_MC_bone_. This may best be explained by the improved depiction of vessel margins, origins, branching and intrahepatic vessel demarcation from surrounding liver tissue found for CACT_MC_no bone_, possibly making this easier to interpret even for less experienced readers. Likewise, the lower kappa values for some of the other categories such as artifact assessment induced by foreign materials in CACT_MC_bone_ or tumor feeder demarcation in CACT_Org_, might in part be explained by differing levels of experience of the readers (8 vs. 1 year). This is underlined not only by the results of the Wilcoxon signed-rank test and McNemar’s test, showing significantly different perceptions for both readers between the datasets but also coincides with the previously published literature of IQ augmentation via motion correction algorithms [18–23], and of course, with the objectively assessed improvement of image sharpness.

## Limitations

A larger study population and a prospective as well as a multi-centric study design might be beneficial in further assessing the potential of the motion correction prototype and its effect on image quality. Due to the retrospective application of the motion correction prototype, we can only make estimations concerning its true impact on the diagnostic process and clinical outcome of patients. An automated process of bone segmentation would ultimately be required to reduce even short waiting times and allow its intra-procedural use. Furthermore, no gold standard exists for the evaluation of CACTs. To this end, the analysis criteria were consensually decided on beforehand by a panel of experts. With motion detection depending upon high-contrast objects, the use of different-sized catheters (macro- and microcatheter) might have an influence on the motion correction algorithm. Potential differences concerning the catheter type were not the focus of the current study and further analyses need to address this issue.

## Conclusion

The application of the dedicated motion correction algorithm was feasible for all data sets and led to a significant increase in both objective and subjective image quality parameters.

## Abbreviations

CACT: C-arm computed tomography
CT: Computer tomography
DSA: Digital subtraction angiography
IQ: Image quality
MC: Motion correction
Org: Original
S: Seconds
Sd: Standard deviation
SIRT: Selective internal radiation therapy
TACE: Transarterial chemoembolization
